# The C-Reactive Protein/Albumin Ratio as an Independent Predictor of Mortality in Patients with Severe Sepsis or Septic Shock Treated with Early Goal-Directed Therapy

**DOI:** 10.1371/journal.pone.0132109

**Published:** 2015-07-09

**Authors:** Min Hyung Kim, Jin Young Ahn, Je Eun Song, Heun Choi, Hea Won Ann, Jae Kyoung Kim, Jung Ho Kim, Yong Duk Jeon, Sun Bean Kim, Su Jin Jeong, Nam Su Ku, Sang Hoon Han, Young Goo Song, Jun Young Choi, Young Sam Kim, June Myung Kim

**Affiliations:** 1 Bundang Jesaeng hospital, Seongam, Gyeonggi, Korea; 2 Department of Internal Medicine Yonsei University College of Medicine, Seoul, Korea; 3 AIDS Research Institue, Yonsei University College of Medicine, Seoul, Korea; Hospital Sirio-Libanes, BRAZIL

## Abstract

**Background:**

Sepsis, including severe sepsis and septic shock, is a major cause of morbidity and mortality. Albumin and C-reactive protein (CRP) are considered as good diagnostic markers for sepsis. Thus, initial CRP and albumin levels were combined to ascertain their value as an independent predictor of 180-day mortality in patients with severe sepsis and septic shock.

**Materials and Methods:**

We conducted a retrospective cohort study involving 670 patients (>18 years old) who were admitted to the emergency department and who had received a standardized resuscitation algorithm (early goal-directed therapy) for severe sepsis and septic shock, from November 2007 to February 2013, at a tertiary hospital in Seoul, Korea. The outcome measured was 180-day all-cause mortality. A multivariate Cox proportional hazard model was used to identify the independent risk factors for mortality. A receiver operating characteristic (ROC) curve analysis was conducted to compare the predictive accuracy of the CRP/albumin ratio at admission.

**Results:**

The 180-day mortality was 28.35% (190/670). Based on the multivariate Cox proportional hazard analysis, age, the CRP/albumin ratio at admission (adjusted HR 1.06, 95% CI 1.03–1.10, p<0.001), lactate level at admission (adjusted HR 1.10, 95% CI 1.05–1.14, p<0.001), and the Sequential Organ Failure Assessment (SOFA) score at admission (adjusted HR 1.12, 95% CI 1.07–1.18, p<0.001) were independent predictors of 180-day mortality. The area under the curve of CRP alone and the CRP/albumin ratio at admission for 180-day mortality were 0.5620 (P<0.001) and 0.6211 (P<0.001), respectively.

**Conclusion:**

The CRP/albumin ratio was an independent predictor of mortality in patients with severe sepsis or septic shock.

## Introduction

Sepsis, including severe sepsis and septic shock, remains a major cause of morbidity and mortality worldwide [1]. The mortality rate of severe sepsis is 20–30% [2–4], accounting for about 30–50% of hospital deaths [5]. Even though the mortality rate of severe sepsis has decreased markedly since the introduction of early resuscitative treatments, including early goal-directed therapy (EGDT), survivors are still at increased risk of death [6, 7]. And reports about elderly suggest survivors of sepsis are at increased risk of death, months and even years after hospital discharge [8].

The inflammatory response is important in the pathophysiology of sepsis, and the impact of inflammation that can worsen chronic illness, which is a major determinant of adverse, long-term outcomes. Thus, biomarkers that can be used as independent prognostic factors to evaluate the mortality of patients with severe sepsis or septic shock should be measured objectively to reflect the inflammatory processes as well as responses to therapeutic intervention. As the level of C-reactive protein (CRP) increases markedly in response to infection, and the magnitude of the increase may correlate with the severity of the infection [9, 10], the prognostic value of CRP levels has been investigated in many diseases [11, 12]. Albumin is also a potent prognostic marker of outcomes in infection-related disease, as its levels decrease during the response to acute phase infections [12, 13]. Based on these properties, we speculated that the ratio of CRP to albumin could be used as a predictive marker for mortality, as has been observed in other diseases [14, 15]. Little is known about the prognostic accuracy of mortality in severe sepsis or septic shock treated with EGDT. We investigated the use of the CRP/albumin ratio as an independent predictor of mortality in these patients where we hypothesized that this ratio could be used as an independent predictor of mortality in patients with severe sepsis or septic shock.

## Materials and Methods

### Ethics statement

The study was approved by the Institutional Review Board (IRB) of Yonsei University Health System Clinical Trial Center. Since the study was retrospective and the study subjects were anonymized, the IRB waived the requirement for written consent from the patients.

### Study population

A retrospective cohort study was conducted, and included adult patients (>18 years) admitted to the emergency department (ED) of Severance Hospital at Yonsei University College of Medicine (Seoul, Korea) with severe sepsis and/or septic shock, from November 2007 to February 2013. Severance Hospital is a 2000-bed tertiary-care teaching hospital. At this institute, EGDT has been implemented in the intensive care unit and the ED since November 2007 as part of a quality improvement initiative. On average, 200 patients a day were screened, and those with two or more systemic inflammatory response syndrome criteria, and signs of infection, were evaluated for EGDT eligibility. One or both of the following conditions triggered initiation of the EGDT protocol: (a) an initial systolic blood pressure of <90 mmHg despite intravenous challenge with 20 ml/kg of crystalloid fluid or (b) an initial serum lactate level of ≥4 mmol/L.

The exclusion criteria for this study included: (a) age <18 years, (b) any contraindication to central venous catheterization, (c) pregnancy, (d) acute cerebrovascular accident, (e) acute coronary syndrome, (f) active gastrointestinal bleeding, (g) trauma, (h) drug overdose, (i) requirement for immediate surgery, (j) absence of informed consent, (k) transfer to another institution, and/or (l) a do-not-resuscitate order.

The resuscitation team was composed of an emergency medicine specialist, a physician and an anaesthesiologist who followed the protocol outlined in the surviving sepsis campaign management guidelines [16]. The team immediately positioned a central venous catheter to monitor pressure and central venous oxygen saturation (ScvO_2_) and to administer intravenous fluids, vasopressors or packed red cell transfusions. The protocol in this study specified crystalloids as the resuscitative fluid and specified norepinephrine as the vasopressor. Patients were resuscitated up to 6 h after enrollment and were evaluated to ascertain whether the goal was achieved. All other aspects of care were administered at the discretion of the treating physician.

### Laboratory measurements

Uniformity of data collection was achieved through implementation of the EGDT protocol [17], which was designed based on the surviving sepsis protocol. A complete blood count (CBC), serum albumin, and CRP and lactate levels of patients were checked at admission to the ED as part of a routine test. ScvO_2_ was checked upon EGDT enrollment, and was monitored again 2, 4, and 6 h afterwards. Serum albumin and CBC levels were monitored daily and CRP was monitored every 3 to 4 d using an automatic nephelometer (Beckman Coulter, Fullerton, CA, USA). Normal ranges were 0.0–0.80 mg/dL for CRP and 35.0–53.0 g/L for albumin. Other laboratory tests, such as a CBC with a red blood cell distribution width (RDW), procalcitonin, lactate, N-terminal pro-hormone brain natriuretic peptide (pro-BNP), and serum chemistry were also performed within 1 h of admission.

### Data collection

Medical records of all patients included were reviewed, and relevant clinical, biological and radiological data were collected. Baseline characteristics, including demographic information and pre-existing chronic comorbidities, were also noted. The comorbidities included diabetes, chronic heart failure, chronic liver disease, chronic pulmonary disease, and malignancy. Initial hemodynamic parameters, including central venous pressure, mean arterial pressure, and ScvO_2_, were also collected. Disease severity was assessed by the worst scores using the acute physiology and chronic health evaluation (APACHE) II, and the sequential organ failure assessment (SOFA) tools, within the first 24 h of ED admission. Additional data such as, time from enrolment to first dose of antibiotics, receipt of mechanical ventilation or whether the patients had received renal replacement therapy, were also noted. The follow-up was evaluated upon the patient’s death or on the last day of the outpatient visit. Mortality was assessed via the hospital records.

### Statistical analysis

Patients lost to follow-up before 180 days were regarded as non-informative censoring. We hypothesized that there was no difference in the baseline characteristics between those who were censored and who were followed up to the end point. In order to explore our hypothesis we conducted statistical analysis comparing baseline characteristics of either group. Baseline characteristics were compared using the Mann-Whitney U test and the independent samples *t*-test for the continuous variables, and the χ^2^ test for categorical variables. Continuous variables were expressed as means **±** standard deviation, or medians (interquartile ranges) and categorical variables as numbers with percentages for the description of baseline characteristics. Sensitivity, specificity, and positive and negative predictive values were calculated using standard methods [18]. Diagnostic performance was compared by the weighted least squares method.

The Cox proportional hazards model was used to determine whether each variable was an independent prognostic factor of mortality. The proportional-hazards assumption was tested by the inclusion of an interaction term between all variables and the natural-log-transformed follow-up time. It was also checked by a log-minus-log survival plot for the categorical variables. We intended to exclude variables that didn’t fit the assumption of proportionality. Covariates for the multivariate Cox model were selected based on a p-value <0.05 in a univariate analysis. Variables that could produce multicollinearity were excluded from the analysis. To fully explore the role of the CRP/albumin ratio as a biomarker in the prediction of 180-day mortality, the ratio was also assessed as a binary variable using the cut off value. Variables were considered significant if p<0.05, and the results were presented as hazard ratios with 95% confidence intervals (CIs). Prediction accuracy was assessed using the area under the receiver operating characteristic (ROC) curve. Cut-off values showing the greatest accuracy were determined using a sensitivity/specificity versus criterion value plot. The patients were categorized into two groups based on this cut-off value. Survival curves were created using the Kaplan-Meier method and compared using the log-rank test. Statistical analyses were performed using SAS version 9.2 (SAS Institute Inc., Cary, NC, USA).

## Results

### Population characteristics

In total, 690 patients with severe sepsis and septic shock were treated with EGDT between November 2007 and February 2013. Among them, 11 patients were excluded due to a lack of data, including CRP and albumin levels at admission. A further 9 patients who did not complete the treatment according to the protocol due to patient refusal were also excluded; therefore, data from 670 patients were analyzed. In total, 167 patients were censored before the full 180 days due to loss of follow up. And 190 (28.35%) died within this period.

The mean age of the enrolled patients was 65.06 ± 14.39, and 352 (52%, 352/670) were male. The median length of follow up of non-survivors was 18 days (0–178). The median SOFA score of the patient was 8(6–10). More than 30% of the patients had diabetes mellitus and more than 30% of the patients had cancer (Table 1).

**Table 1:** Baseline features of the enrolled patients.

	Total
**Characteristic**	
Age (years)	65.06±14.39
Male Gender	352(52.54)
**Laboratory Analysis**	
CRP (mg/dL)^1^	15.22±1.12
Albumin (g/L)	31.41±7.17
CRP^1^/albumin ratio	5.298±4.258
CRP (mg/dL)^1^ at 72 h ^a^	11.56±10.02
Albumin (g/L) at 72 h ^b^	27.01±5.57
CRP^1^/albumin ratio at 72 h ^c^	4.58±4.15
Procalcitonin (ug/L) ^d^	35.75±53.52
WBC (x10^3^/mm^3^)^2^	13221.47±10568.73
BUN (mg/dL)^3^	34.34±28.43
Cr (mg/dL)^4^	2.10±1.87
Lactate (mmol/L) ^e^	4.14±3.46
**Rate of goals achieved**	
CVP^5^	524/670 (78.21)
MAP^6^	646/670 (96.41)
ScvO_2_ ^7^	574/670 (85.67)
**Comorbidities**	
DM^8^	215/670(32.09)
COPD^9^	64/670(9.55)
CHF^10^	68/670(10.15)
CRF^11^	69/670(10.31)
Chronic liver disease	64/670(9.55)
Malignancy	233/670(37.89)
SOFA score^12^	8(6–10)
**Time from enrollment to first dose of antibiotics (minutes)**	117.96±130.26

The data was divided into two groups according to survival status at 180 days. Data are expressed as the mean ± SD / median (Q1-Q3) or N (%).

Abbreviations

^1^CRP, C-reactive protein

^2^WBC, white blood cell count

^3^BUN, blood urea nitrogen

^4^Cr, creatinine

^5^CVP, central venous pressure (Goal of CVP in EGDT protocol: 8-12mm Hg)

^6^MAP, mean arterial pressure(Goal of CVP in EGDT protocol: >65 and ≤90mm Hg) and

^7^SCvO_2_, central venous oxygen saturation(Goal of CVP in EGDT protocol: >70%)

^8^DM, diabetes mellitus

^9^ COPD, chronic obstructive pulmonary disease

^10^CHF, congestive heart failure

^11^CRF, chronic renal failure

^12^SOFA, sequential organ failure assessment.

^a^ the number of patients who had available data was 524

^b^ the number of patients who had available data was 596

^c^ the number of patients who had available data was 496

^d^ the number of patients who had available data was 148

^e^ the number of patients who had available data was 663.

### The CRP/albumin ratio as an independent predictor of 180-day all-cause mortality

In a univariate Cox proportional hazard analysis, age, gender, SOFA score, malignancy, CRP, albumin, the CRP/albumin ratio at admission and CRP/albumin ratio at 72 h after admission were significantly associated with 180-day mortality. Furthermore, in a multivariate Cox proportional hazard analysis, age (p = 0.001), SOFA score (p<0.001), lactate level (p<0.001), and CRP/albumin ratio (p<0.001) at admission were independent predictors of mortality. The hazard ratio of 180-day mortality for severe sepsis patients with an elevated CRP/albumin ratio was 1.06 (95% CI, 1.03–1.10), that for an elevated SOFA score was 1.12 (95% CI, 1.07–1.180), that for advanced age was 1.02(95% CI, 1.01–1.03), and the ratio for patients with elevated lactate levels was 1.10 (95% CI, 1.05–1.14) (Table 2).

**Table 2:** Cox proportional hazards analysis for 180-day mortality (CRP/albumin ratio at admission).

Variable	Univariate HR^1^	95% CI	*p*-value	Multivariate HR	95% CI	*p*-value
Age (per 1-y increase)	1.02	1.01–1.03	**0.006**	1.02	1.01–1.03	**0.001**
Gender						
Male	1					
Female	0.64	0.48–0.86	**0.003**	0.90	0.66–1.22	0.48
SOFA score	1.00	1.000–1.003	**<0.001**	1.12	1.07–1.180	**<0.001**
CRP at admission	1.002	1.001–1.004	**0.01**			
Albumin at admission	0.90	0.88–0.92	**<0.001**			
CRP/albumin at admission	1.04	1.00–1.08	**<0.001**	1.06	1.03–1.10	**<0.001**
Lactate at admission	1.14	1.11–1.18	**<0.001**	1.10	1.05–1.14	**<0.001**
Malignancy	1.76	1.31–2.36	**0.001**	1.24	0.79–1.95	0.344

^1^The hazard ratio (HR) for death is expressed per 1-y increase in age, female to male in gender, per 1 unit increase in the SOFA score, 1 mg/L increase in CRP, 1 g/dL decrease in albumin, 1 increase in CRP (mg/dL)/albumin(g/dL), 1 mmol/dL increase in lactate and having malignancy to having no malignancy.

The multivariate analysis was adjusted for covariates such as age, gender, CRP/albumin ratio, SOFA score, lactate level and having malignancy or not, for each variable. Abbreviations: SOFA, sequential organ failure assessment; CRP, C-reactive protein

A second model to predict 180-day mortality was also created. This model included the CRP/albumin ratio at 72 h after admission rather than the CRP/albumin ratio at admission. Fifty-eight patients who died within 72 h, and 116 patients with missing biomarkers at 72 h among those not confirmed to be dead were excluded from this analysis. In this model, age, SOFA score and lactate level were independent predictors of 180-day mortality but the CRP/albumin ratio at 72 h after admission was not an independent predictor (Table 3).

**Table 3:** Cox proportional hazards analysis for 180-day mortality (CRP/albumin ratio at 72 h after admission).

Variable	Univariate HR^1^	95% CI	*p*-value	Multivariate HR	95% CI	*p*-value
Age (per 1-y increase)	1.02	1.01–1.03	0.006	1.02	1.00–1.03	**0.020**
Gender						
Male	1					
Female	0.64	0.48–0.86	**0.003**	0.70	0.48–1.03	0.067
SOFA score	1.197	1.15–1.25	**<0.001**	1.12	1.06–1.19	**0.001**
CRP at 72 h	1.00	1.00–1.003	0.176			
Albumin at 72 h	0.86	0.83–0.9	**<0.001**			
CRP/albumin at 72 h	1.08	1.05–1.11	**<0.001**	1.02	0.98–1.06	0.348
Lactate at admission	1.14	1.11–1.18	**<0.001**	1.06	1.01–1.12	**0.027**
Malignancy	1.76	1.31–2.36	**0.001**	1.19	0.71–2.00	0.512

^1^The hazard ratio (HR) for death is expressed per 1-y increase in age, female to male in gender, per 1 score increase in the SOFA score, 1 mg/L increase in CRP, 1 g/L decrease in albumin, 1 increase in CRP(mg/dL)/albumin(g/L), 1 mmol/dL increase in lactate and having malignancy to having no malignancy.

The multivariate analysis was adjusted for covariates such as age, gender, CRP/albumin ratio, SOFA score, lactate level and having malignancy or not, for each variable. Abbreviation: SOFA, sequential organ failure assessment; CRP, C-reactive protein.

### Diagnostic performance of the CRP/albumin ratio

**Fig 1:**
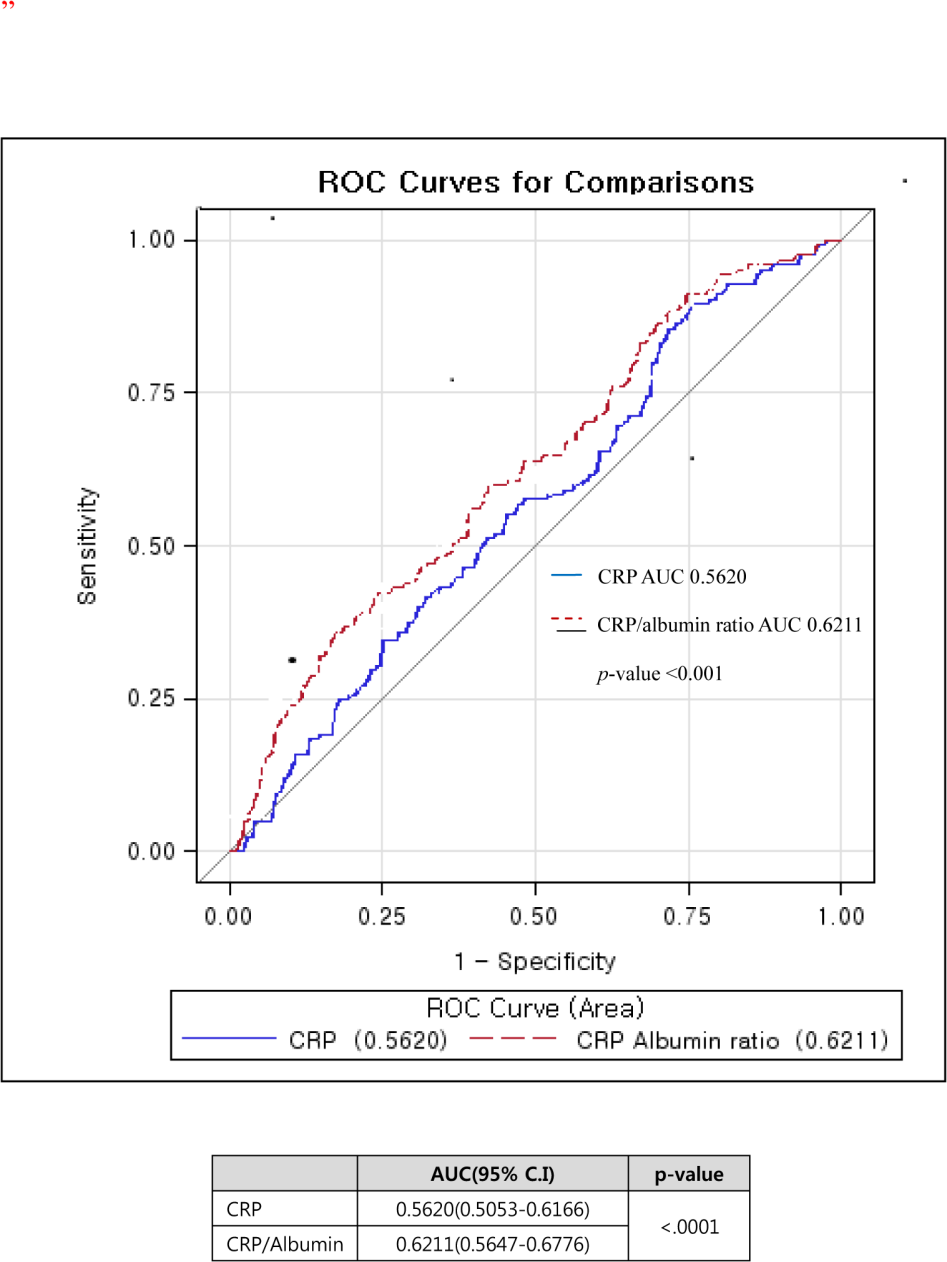
Receiver operating characteristic (ROC) curve of CRP and CRP/albumin ratio in predicting 180-day mortality. The area under curve (AUC) for the CRP/albumin ratio is higher compared to CRP alone (p<0.001).

**Fig 2:**
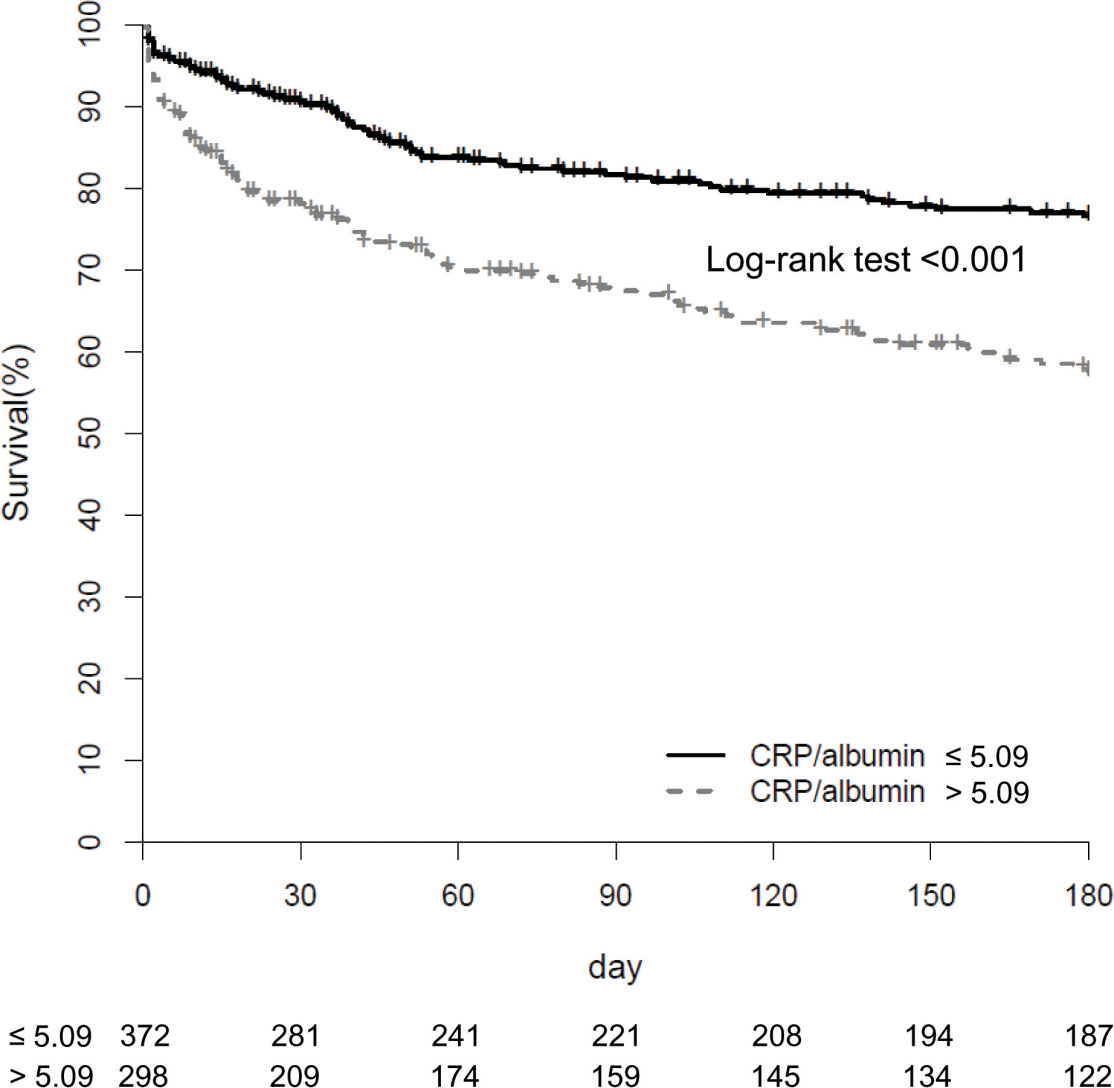
Kaplan-Meier analysis of the cumulative percentage of surviving patients at 180-days according to the different CRP/albumin levels. Patients with higher levels of CRP/albumin ratio had a reduced survival rate. Solid line, CRP/albumin ratio ≤5.09; dashed line, CRP/albumin ratio >5.09.

The prediction of mortality was assessed using the area under the ROC curve (AUC) (Fig 1). The AUC for the CRP/albumin ratio at admission (0.62 [95% CI, 0.56–0.68; p<0.001]) was greater than that for CRP alone (0.56 [95% CI, 0.51–0.62; p<0.001]). Moreover, a mountain curve analysis determined the most sensitive and specific cut-off value for CRP to be 6.75 mg/dL; the cut-off for the CRP/albumin ratio was 5.09 (S1 Fig). In addition, the sensitivity and specificity of the CRP/albumin ratio cut-off value (>5.09) were 61 and 61%, respectively, while the CRP cut-off value was less specific (sensitivity and specificity of 84 and 31%, respectively) (Table 4). Analysis using the CRP/albumin ratio as the binary variable resulted in a hazard ratio of 180-day mortality with an elevated CRP/albumin ratio of 1.74 (95% CI, 1.27–2.37) (S1 Table). A Kaplan-Meier curve analysis also showed a significantly higher 180-day mortality rate in patients with a CRP/albumin ratio >5.09, compared to patients with a lower CRP/albumin ratio (Fig 2).

**Table 4:** Comparison of the diagnostic performance of each factor in predicting 180-day mortality.

Day 180	TN	FN	FP	TP	Cut-off point	Sensitivity	Specificity	Accuracy	PPV	NPV
CRP at admission	147	28	328	157	>67.5	84.86(79.70–90.03)	30.95(26.79–35.10)	46.06(42.26–49.86)	32.37(28.21–36.53)	84.00(78.56–89.43)
CRP/Albumin at admission	290	72	185	113	>5.09	61.08(54.06–68.11)	61.05(56.67–65.44)	61.06(57.34–64.78)	37.92(32.41–43.43)	80.11(76.00–84.22)
CRP at 72 h	130	29	263	99	>47.13	77.34(70.09–84.60)	33.08(28.43–37.73)	43.95(39.69–48.22)	27.34(22.76–31.94)	81.76(75.76–87.76)
CRP/Albumin at 72 h	84	15	285	112	>0.91	88.19(82.58–93.80)	22.76(18.49–27.04)	39.51(35.21–43.82)	28.21(23.78–32.63)	84.85(77.79–91.91)

Figures in brackets indicate the 95% confidence intervals of each value. Figures in TN, FN, FP, and TP indicate the numbers of patients included in each group. Abbreviations: CRP, C-reactive protein; TN, true negative; FN, false negative; FP, false positive; TP, true positive; PPV, positive predictive value; NPV, negative predictive value.

## Discussion

With the success of initial resuscitation treatments and decreasing death rates for severe sepsis or septic shock, the focus has moved to the later prognosis of the patients. Our study shows that the CRP/albumin ratio at admission can be used as an independent predictor of 180-day mortality in patients with severe sepsis or septic shock. To our knowledge, few studies have evaluated the prognostic significance of the CRP/albumin ratio in patients treated with EGDT, whereas many studies have investigated the use of inflammatory markers, such as CRP, procalcitonin, and pro-BNP to predict mortality in critically ill patients [13, 19, 20]. Among these markers, CRP is readily accessible in the critical care setting, and its value as a prognostic marker is proven in many diseases, including cerebral disease, heart failure, and sepsis [11, 12, 21]. However, these studies mainly focused on 28- to 30-day mortality. In our study, the end point was 180-day mortality and the significance of CRP was evaluated as a prognostic factor in severe sepsis and septic shock patients. A high level of CRP correlated with 180-day mortality, but with an only modest to low predictive capability. Despite previous studies that investigated the relevance of CRP in cases of poor prognosis, its predictive value has not been confirmed. Thiem et al. [22] reported that for hospitalized patients with community-acquired pneumonia, the initial CRP level did not predict mortality. In another large prospective study of nonsurgical intensive care patients, plasma CRP measured at the day of discharge from intensive care did not predict re-admission or death [23].

Albumin levels are associated with the chronic nature of disease, and represent the inflammatory status [24, 25]. The value of albumin levels in predicting outcomes in chronic and inflammatory disease is also well known [12, 13, 26]. In patients with community-acquired bloodstream infections, with severe sepsis or septic shock, hypoalbuminemia is the strongest predictor of mortality [13]. However, in the context of this study, hypoalbuminemia is likely to be the result of infection rather than previous illness. As such, when evaluating patients with varying underlying conditions, this marker alone can create bias because albumin levels are affected by both the chronic nutritional and inflammatory status.

It is generally believed that the worsening of a chronic illness with an ongoing infection represents a major determinant of an adverse long-term outcome in severe sepsis and septic shock patients. Thus, rather than an analysis of each single factor on its own, CRP and albumin were combined. The combination of these markers, enabled inflammatory and nutritional factors to be merged, both of which strongly influence prognosis [14]. Furthermore, the CRP/albumin ratio may be an indicator of a stronger inflammation response. In this study, the CRP/albumin ratio at admission was positively correlated with the prognosis in severe sepsis and septic shock patients treated with EGDT. Ranzani et al. [15] had already demonstrated that the CRP/albumin ratio, which indicates the extent of residual inflammation, could be used as a prognostic marker of 90-day mortality after discharge in sepsis patients. However, in that study only 33% of the patients had severe sepsis and septic shock, and only those patients who were discharged alive were included. In contrast, this study included more-severe patients, of which 18% (124/670) died before they could be discharged. Our findings showed that the CRP/albumin ratio was able to predict prognosis even in this smaller group of patients. A CRP/albumin ratio >5.09 had the best sensitivity and specificity in predicting 180-day mortality. There was a difference between non survivors and the rest in the proportion of patients in terms of malignancy and age, but even after adjusting for these factors, the CRP/albumin ratio still influenced prognosis at a significant level. Although the AUC for the CRP/albumin ratio was only 0.62 (95% CI, 0.56–0.68), indicating a modest predictive capability, the survival difference depicted in the Kaplan-Meier curve analysis supported the usefulness of this model. Our results showed that the CRP/albumin ratio before treatment was a predictor of prognosis. The importance of the pretreatment values was also observed in other studies that showed that initial clinical measurements were often the best predictors of physiological perturbations. Specific prognostic capabilities have been shown for a number of biomarkers, including lactate, heart rate, and autonomic dysfunction [27–29]. Taken together with these markers, the CRP/albumin ratio at admission can be used to stratify patients according to the severity of disease, even after the patients have been discharged from hospital.

In this study, patients were treated with EGDT. The goal achievement rate of all three parameters (CVP 8-12mm Hg, MAP>65 and ≤90mm Hg, ScvO_2_≥70%) was 73.45%. In this study, we evaluated whether changes in CRP, albumin, or the CRP/albumin ratio after 72 h would affect the patient prognosis after the resuscitative procedure; only 496 patients with available data at 72 h were included. No statistical significance was observed for the CRP/albumin ratio at 72 h in patient prognosis. Our results may have been affected by the fact that the more critically ill patients who died within 72 h were excluded from the analysis. However, considering that the CRP/albumin ratios were still high for both groups, the result may be interpreted as follows: Because the plasma half-life of CRP is ~19 h when the entire stimulus for increased production completely ceases [30] while albumin levels remain constant during that short time period of time, the CRP/albumin ratio at 72 h may have been mainly affected by the CRP value. This value may represent residual inflammation immediately after resuscitation. However, as the value at 72 h was not significant, this demonstrated that the CRP/albumin ratio at 72 h may not be used as a tool to guide decisions on the treatment of patients with severe sepsis and septic shock. This is consistent with the result of a previous study of CRP values, which showed that at 2 d after treatment, the values were not significantly different between survivors and non-survivors [31].

Well-established biomarkers, including lactate levels and SOFA scores, were used as independent predictors in our analyses. These scores are strongly associated with organ dysfunction, and are frequently used to guide therapeutic decisions and allocate resources [32, 33]. The contribution of changes in each of these values to the prognosis of severe sepsis and septic shock patients should be evaluated on a larger scale.

There were several limitations to our study. First, as this was a retrospective study, possible bias was likely. Second, the variables were collected from a single center, making generalization of these results to other institutions difficult. Additional prospective studies with larger populations involving multiple centers are necessary to accurately evaluate the CRP/albumin ratio as a predictor of mortality.

In conclusion, the CRP/albumin ratio is an independent predictor of long-term mortality in patients with severe sepsis or septic shock.

## Supporting Information

S1 Fig Plot versus criterion value curves for the CRP versus 180-day mortality and the CRP/albumin ratio versus 180-day mortality. The X-axis shows the CRP level in mg/L and CRP/albumin ratio. The Y-axis shows the percentage. The solid and dashed lines indicate sensitivity and specificity with 95% confidence intervals, respectively.
(TIF)

S1 Table Cox proportional hazards analysis for 180-day mortality (CRP/albumin ratio at admission as a categorical variable). (DOCX)
