# Correction: The C-reactive protein/albumin ratio as an independent predictor of mortality in patients with severe sepsis or septic shock treated with early goal-directed therapy

**DOI:** 10.1371/journal.pone.0225620

**Published:** 2019-11-18

**Authors:** Min Hyung Kim, Jin Young Ahn, Je Eun Song, Heun Choi, Hea Won Ann, Jae Kyoung Kim, Jung Ho Kim, Yong Duk Jeon, Sun Bean Kim, Su Jin Jeong, Nam Su Ku, Sang Hoon Han, Young Goo Song, Jun Young Choi, Young Sam Kim, June Myung Kim

An additional affiliation for the first author is not indicated. Min Hyung Kim is also affiliated with: Department of Internal Medicine Yonsei University College of Medicine, Seoul, Korea.

## References

[1] Kim MH, Ahn JY, Song JE, Choi H, Ann HW, Kim JK, et al (2015) The C-reactive protein/albumin ratio as an independent predictor of mortality in patients with severe sepsis or septic shock treated with early goal-directed therapy. PLoS ONE 10(7): e0132109 10.1371/journal.pone.0132109 26158725 PMC4497596

