# Supplementary figures and images for: The C-Reactive Protein/Albumin Ratio as an Independent Predictor of Mortality in Patients with Severe Sepsis or Septic Shock Treated with Early Goal-Directed Therapy
Source: PLoS One. 2015 Jul 9;10(7):e0132109. doi: 10.1371/journal.pone.0132109 (PMC4497596; doi:10.1371/journal.pone.0132109)

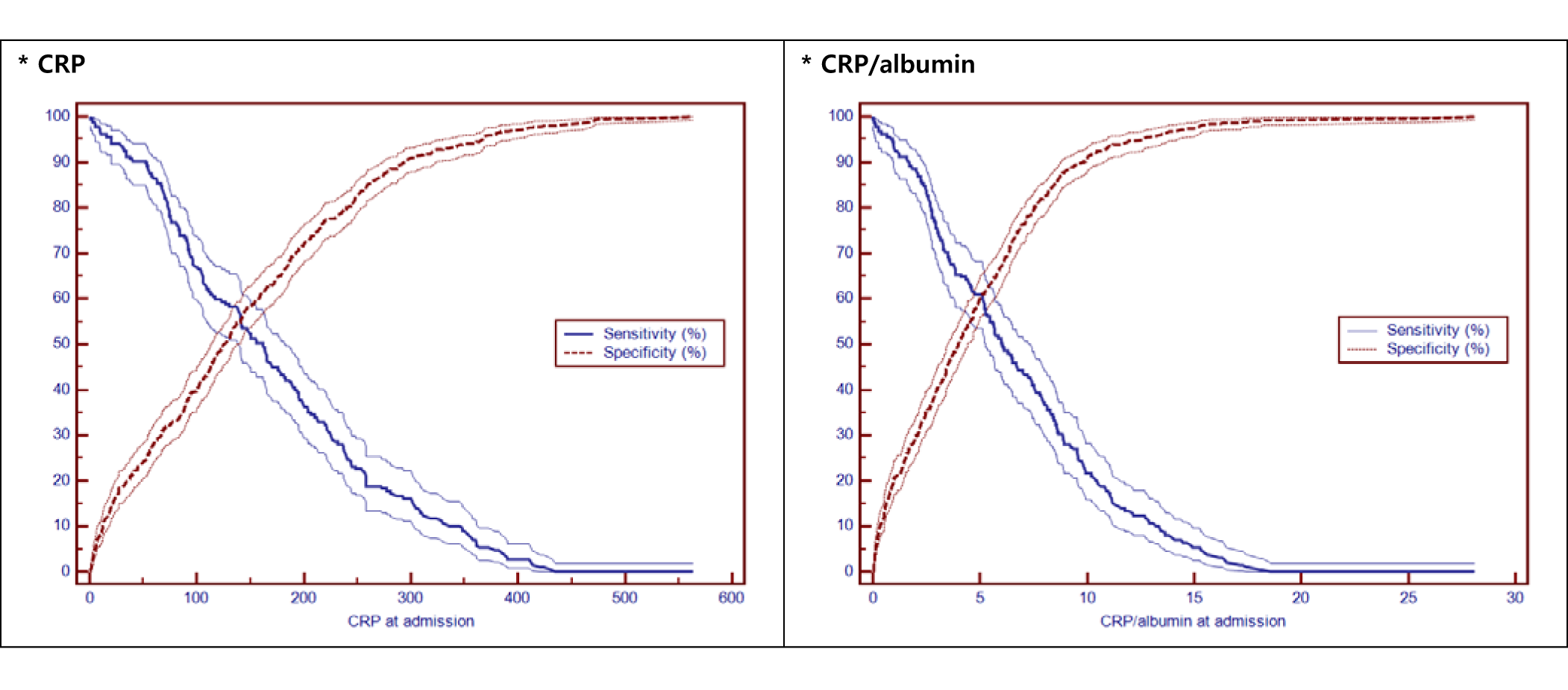

Supplement: S1 Fig — The X-axis shows the CRP level in mg/L and CRP/albumin ratio. The Y-axis shows the percentage. The solid and dashed lines indicate sensitivity and specificity with 95% confidence intervals, respectively. (TIF) [file pone.0132109.s001.tif]
