# Supplementary material for: The C-Reactive Protein/Albumin Ratio as an Independent Predictor of Mortality in Patients with Severe Sepsis or Septic Shock Treated with Early Goal-Directed Therapy
Source: PLoS One. 2015 Jul 9;10(7):e0132109. doi: 10.1371/journal.pone.0132109 (PMC4497596; doi:10.1371/journal.pone.0132109)
Supplement: S1 Table — (DOCX) [file pone.0132109.s002.docx]

**S1 table. Cox proportional hazards analysis for 180-day mortality (CRP/albumin ratio at admission as a categorical variable)**

| **Variable** | **Univariate HR^１^** | **95% CI** | ***p-*value** | **Multivariate HR** | **95% CI** | ***p-*value** |
| --- | --- | --- | --- | --- | --- | --- |
| Age (per 1-y increase) | 1.02 | 1.01-1.03 | **0.006** | 1.02 | 1.07-1.03 | **0.002** |
| Gender |  |  |  |  |  |  |
| Female | 1 |  |  | 1 |  |  |
| Male | 0.64 | 0.48-0.86 | **0.003** | 0.90 | 0.66-1.22 | 0.496 |
| SOFA score | 1.00 | 1.000-1.003 | **<0.001** | 1.12 | 1.07-1.18 | **<0.001** |
| CRP/albumin at admission |  |  |  |  |  |  |
| CRP/Albumin ≤5.09 | 1 |  |  | 1 |  |  |
| CRP/Albumin >5.09 | 2.07 | 1.54-2.78 | **<0.001** | 1.74 | 1.27-2.37 | **<0.001** |
| Lactate at admission | 1.14 | 1.11-1.18 | **<0.001** | 1.10 | 1.05-1.14 | **<0.001** |
| Malignancy | 1.76 | 1.31-2.36 | **0.001** | 1.25 | 0.80-1.96 | 0.332 |

^１^The hazard ratio (HR) for death is expressed per 1-y increase in age, female to male in gender, per 1 unit increase in the SOFA score, 1 mg/L increase in CRP, 1 g/dL decrease in albumin, 1 increase in CRP (mg/dL)/albumin(g/dL), 1 mmol/dL increase in lactate and having malignancy to having no malignancy.

The multivariate analysis was adjusted for covariates such as age, gender, CRP/albumin ratio, SOFA score, lactate level and having malignancy or not, for each variable. Abbreviations: SOFA, sequential organ failure assessment; CRP, C-reactive protein
